# *SMURF1* Amplification Promotes Invasiveness in Pancreatic Cancer

**DOI:** 10.1371/journal.pone.0023924

**Published:** 2011-08-22

**Authors:** Kevin A. Kwei, A. Hunter Shain, Ryan Bair, Kelli Montgomery, Collins A. Karikari, Matt van de Rijn, Manuel Hidalgo, Anirban Maitra, Murali D. Bashyam, Jonathan R. Pollack

**Affiliations:** 1 Department of Pathology, Stanford University, Stanford, California, United States of America; 2 Department of Pathology, The Johns Hopkins University, Baltimore, Maryland, United States of America; 3 Department of Oncology, The Johns Hopkins University, Baltimore, Maryland, United States of America; 4 Clinical Research Program, Centro Nacional de Investigaciones Oncológicas, Madrid, Spain; 5 Laboratory of Molecular Oncology, Centre for DNA Fingerprinting and Diagnostics, Nampally, Hyderabad, India; Technische Universität München, Germany

## Abstract

Pancreatic cancer is a deadly disease, and new therapeutic targets are urgently needed. We previously identified DNA amplification at 7q21-q22 in pancreatic cancer cell lines. Now, by high-resolution genomic profiling of human pancreatic cancer cell lines and human tumors (engrafted in immunodeficient mice to enrich the cancer epithelial fraction), we define a 325 Kb minimal amplicon spanning *SMURF1*, an E3 ubiquitin ligase and known negative regulator of transforming growth factor β (TGFβ) growth inhibitory signaling. *SMURF1* amplification was confirmed in primary human pancreatic cancers by fluorescence *in situ* hybridization (FISH), where 4 of 95 cases (4.2%) exhibited amplification. By RNA interference (RNAi), knockdown of SMURF1 in a human pancreatic cancer line with focal amplification (AsPC-1) did not alter cell growth, but led to reduced cell invasion and anchorage-independent growth. Interestingly, this effect was not mediated through altered TGFβ signaling, assayed by transcriptional reporter. Finally, overexpression of SMURF1 (but not a catalytic mutant) led to loss of contact inhibition in NIH-3T3 mouse embryo fibroblast cells. Together, these findings identify *SMURF1* as an amplified oncogene driving multiple tumorigenic phenotypes in pancreatic cancer, and provide a new druggable target for molecularly directed therapy.

## Introduction

Pancreatic ductal adenocarcinoma (hereafter, pancreatic cancer) is nearly always fatal, with a five year survival rate less than 5% [1]. It is often disseminated at diagnosis, and can metastasize widely. Early detection can improve survival, but surgical resection is rarely curative [2]. Pancreatic cancer is also largely resistant to conventional chemotherapy. Therefore, new therapies are urgently needed. In particularly, it will be important to discover and validate new targets for molecularly-directed therapy.

The molecular genetics of pancreatic cancer are in part known [3], [4]. Somatic activating mutations of *KRAS* (sometimes occurring with gene amplification) are found in >90% of pancreatic cancers. Also common are inactivating mutations/deletions of tumor suppressors *CDKN2A* (>95% of cancers), *TP53* (50–75%), and *SMAD4* (also known as *DPC4*) (55%), an effector of TGFβ-mediated growth inhibition. Other gene mutations, each occurring in less than 5% of cancers, impact these and other core cancer signaling pathways [5].

Genomic profiling studies, by array-based comparative genomic hybridization (array CGH), have begun to catalogue DNA amplifications and deletions, pinpointing and revealing novel pancreatic cancer genes (e.g. [6], [7]). Among altered loci, we and others previously identified 7q21-q22 as a site recurrently amplified in pancreatic cancer [8]–[13]. Here, we narrow that locus, and characterize SMURF1 as an oncogene product promoting cell invasion and anchorage-independent growth.

## Results

### *SMURF1* is focally amplified in pancreatic cancer

We had previously identified recurrent amplification at 7q21-q22 in pancreatic cancer cell lines, using CGH on cDNA microarrays [12]. To further delimit the amplicon, and pinpoint the resident oncogene(s), we now carried out additional genomic profiling of a collection of 22 pancreatic cancer cell lines and 58 early-passage pancreatic cancer xenografts, using high-resolution 244K Agilent CGH arrays. The 7q21-q22 locus was focally amplified (tumor/normal ratios >3-fold) in 1 of 22 (4.5%) cell lines (AsPC-1), and in 1 of 58 (2%) xenografts. Including lower-level gains (ratios >1.3 fold), gain/amplification spanning 7q21-q22 was found in 6 of 22 (27%) cell lines, and in 19 of 58 (33%) xenografts.

**Figure 1:**
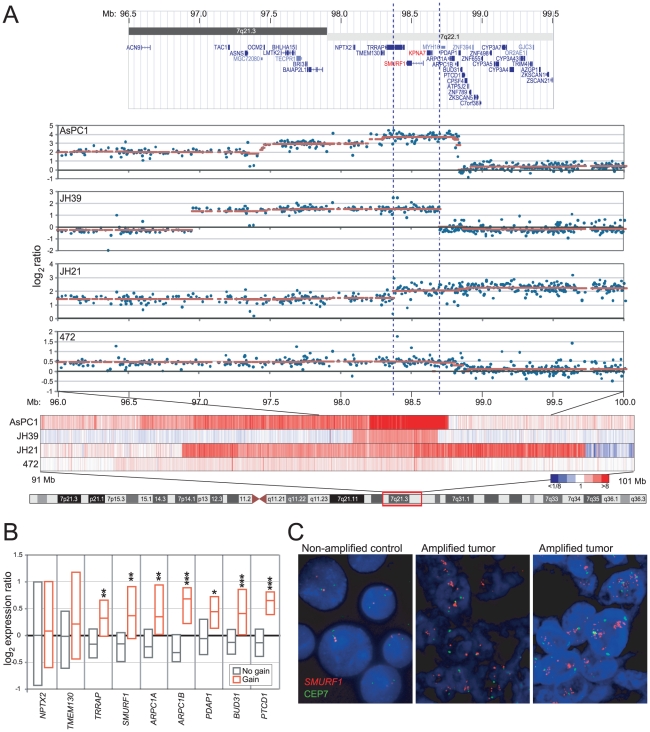
Focal amplification of 7q22.1 in pancreatic cancer spans *SMURF1*. (**A**) A minimal amplicon is defined by four pancreatic cancer specimens (AsPC-1 and three xenografts). Starting from bottom: chr 7 ideogram; Heatmap of DNA copy number (red indicates gain) for the four specimens across the 7q21-q22 region (91–101 Mb); Scatter plot of DNA copy number log_2_ ratios across 7q21.3-q22.1 (96–100 Mb), overlaid with the cghFLasso [34] called ratios (red line); Screen shot of the corresponding locus from the UCSC genome browser. The dashed lines bracket the 325 Kb minimal amplicon, which spans *SMURF1*. (**B**) *SMURF1* is overexpressed when gained/amplified. Box plots show 25^th^, 50^th^ (median) and 75^th^ percentile transcript levels (assayed by Agilent 44K array) for specimens with (red) or without (gray) 7q21-q22 gain, for *SMURF1* and its nearest gene neighbors. Note, *KPNA7* was not represented on the array. *, *P*<0.05; **, *P*<0.01; ***, *P*<0.001 (Mann-Whitney U-test). (**C**) FISH reveals *SMURF1* amplification in primary pancreatic cancers. Shown are two pancreatic cancers with *SMURF1* amplification (*center*, and *right*), along with a non-amplified control (BxPC3 cells, *left*). *SMURF1* locus probe (red); control chr 7 centromere (CRP7) probe (green).

Four specimens (the AsPC-1 cell line, and three xenografts) had genomic profiles that were particularly informative in delimiting the amplicon boundaries within 7q21-q22 (Fig. 1A). The smallest common region of gain spanned just 325 Kb within cytoband 7q22.1, and contained just two RefSeq [14] genes, *SMURF1* (SMAD specific E3 ubiquitin protein ligase) and *KPNA7* (karyopherin alpha 7). SMURF1 is a known inhibitor of TGFβ signaling (by promoting degradation of its receptor TGFβRI, and signaling mediator SMAD4 [15], [16]), a pathway frequently disrupted in pancreatic cancer. Given an obvious connection to pancreatic carcinogenesis, we therefore focused subsequent efforts on *SMURF1*.

Consistent with an oncogenic role, *SMURF1* transcript (measured by microarray) was significantly elevated in cell lines/xenografts with 7q22.1 gain/amplification (as were several co-amplified neighboring genes) (Fig. 1B). To evaluate *SMURF1* amplification in primary pancreatic tumors, we also carried out FISH on a tissue microarray containing 105 pancreatic cancer cases. Four of 95 (4.2%) evaluable cases exhibited *SMURF1* amplification (locus/centromere ratio >2.5) (Fig. 1C), comparable to our CGH findings for early-passage xenografts. We were unable to identify a suitable antibody and staining conditions to evaluate SMURF1 expression by immunohistochemistry.

### *SMURF1* amplification promotes cell invasion and anchorage-independent growth

**Figure 2:**
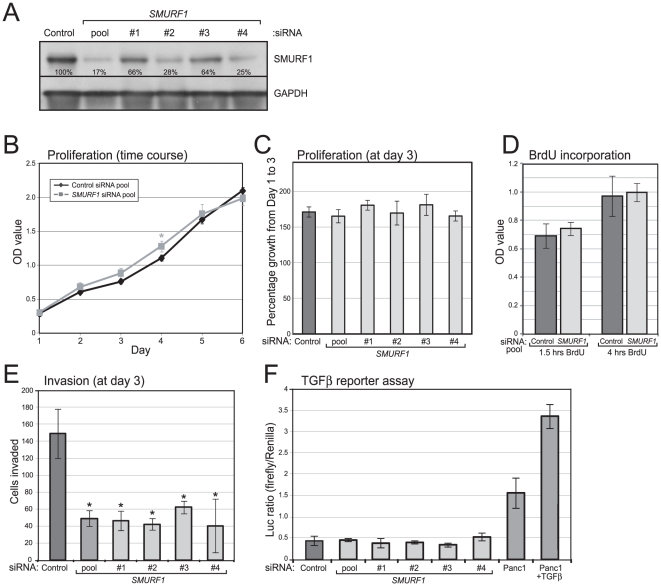
SMURF1 knockdown in amplified AsPC-1 cells reduces invasion but not growth. (**A**) Four different siRNAs targeting *SMURF1*, and a pool of all four, lead to reduced SMURF1 levels (by Western blot) compared to a non-targeting control siRNA pool). Residual SMURF1 levels, here normalized to GAPDH, are indicated. (**B**) SMURF1 knockdown (using siRNA pool) does not reduce cell proliferation/viability, measured by WST1 assay, and done in triplicate (mean +/− 1SD shown). (**C**) SMURF1 knockdown using four different siRNAs does not significantly alter cell proliferation/viability, measured three days post transfection. Done in triplicate (mean +/− 1SD shown). (**D**) SMURF1 knockdown does not reduce cell-cycle progression (S-phase), measured by BrdU incorporation (1.5 hr and 4 hr pulse labeling), done in triplicate (mean +/− 1 SD shown). (**E**) SMURF1 knockdown (using siRNA pool and individual siRNAs) inhibits cell invasion through Matrigel. Boyden chamber assay done in triplicate and harvested three days post transfection (mean +/− 1SD shown); *, *P*<0.05 (Student's t-test). (**F**) SMURF1 knockdown does not enhance TGFβ pathway-mediated transcription. AsPC-1 cells were co-transfected with siRNAs and p3TP-Lux reporter, done in triplicate, and firefly/Renilla luciferase ratios shown (mean +/− 1SD shown). Panc1 cells (with wildtype *SMAD4*) +/− TGFβ serve as a positive control.

To evaluate possible oncogenic functions of SMURF1, we first used RNAi to knockdown SMURF1 expression in the relevant context of gene amplification, using AsPC-1 cells. Transfection of four different small interfering RNAs (siRNAs), or a pool of the four together, each led to reduced SMURF1 protein levels (by Western blot), compared to a non-targeting siRNA pool (Fig. 2A). Knockdown of SMURF1 did not alter cell proliferation, measured by WST-1 assay (Fig. 2B,C), and by BrdU incorporation (Fig. 2D), but led to significantly decreased cell invasion through Matrigel, measured by Boyden chamber assay (Fig. 2E). Decreased invasion was seen with each of the four siRNAs targeting distinct *SMURF1* sequences, while the growth rate of the cells remained unchanged within the same time period (Fig. 2C), strongly supporting the specific role of SMURF1 in the invasiveness phenotype.

Given its known, antagonistic function in the TGFβ pathway, we also sought to evaluate the effect of SMURF1 knockdown on TGFβ signaling, using a TGFβ responsive transcriptional reporter (p3TP-Lux) [17]. Knockdown of SMURF1 did not enhance TGFβ pathway-mediated transcription in AsPC-1 cells (Fig. 2F). Of note, however, AsPC-1 cells (like most pancreatic cancers) harbor a mutated *SMAD4*, here SMAD4 (R100T) [18], characterized to be inactivating [19], [20]. Therefore, AsPC-1 cells are likely incapable of a TGFβ pathway transcriptional response. More generally, these findings suggest that the main effect(s) of *SMURF1* amplification/overexpression are likely mediated through pathways distinct from TGFβ signaling.

**Figure 3:**
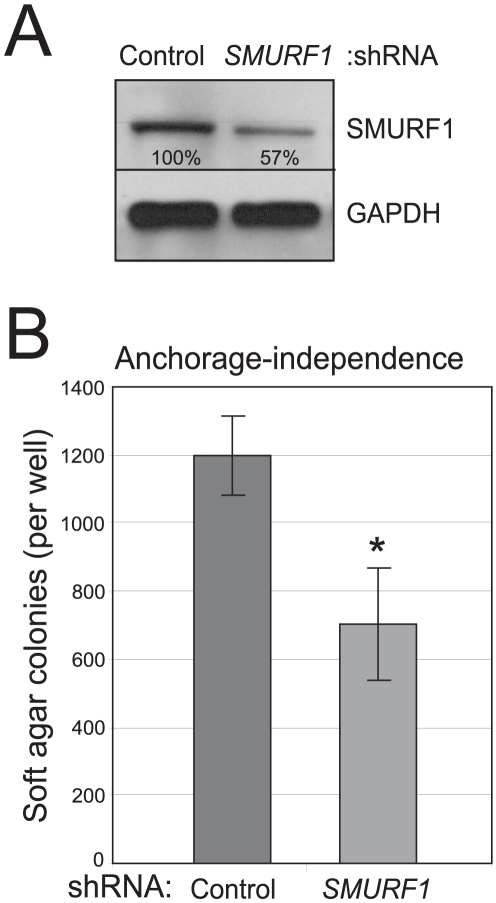
Knockdown of amplified *SMURF1* in AsPC-1 cells reduces anchorage-independent growth. (**A**) A stably transduced shRNA targeting SMURF1 leads to reduced SMURF1 levels (by Western blot) compared to a non-targeting control shRNA. Residual SMURF1 levels, here normalized to GAPDH, are indicated. (**B**) Stable SMURF1 knockdown reduces anchorage-independent growth (i.e. soft agar colonies). Assay done in triplicate (mean +/− 1SD shown); *, *P*<0.05 (Student's t-test).

To evaluate longer-term phenotypes, we also stably transfected a short hairpin RNA (shRNA) targeting *SMURF1*. Stable knockdown of SMURF1 in AsPC-1 cells, confirmed by Western blot (Fig. 3A), significantly reduced anchorage independent growth (soft agar colonies), compared to a non-targeting shRNA control (Fig. 3B).

**Figure 4:**
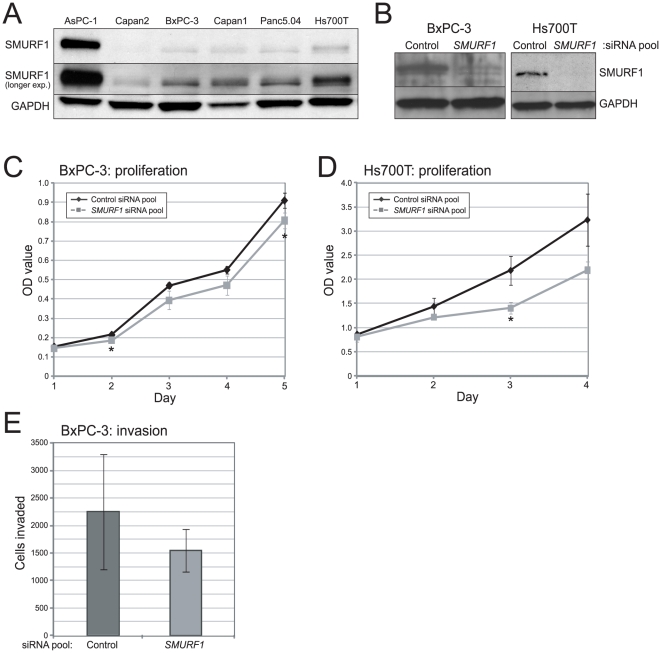
Analysis of SMURF1 knockdown in other pancreatic cancer lines. (**A**) Western blot analysis of endogenous SMURF1 protein levels in a panel of pancreatic cancer cell lines. Note that SMURF1 is highly expressed only in the 7q22.1-amplified AsPC-1 cell line. Two different exposures of the SMURF1 blot are shown; GAPDH serves as a loading control. (**B**) Western blot verification of SMURF1 knockdown (by SMURF1-targeting siRNA pool, compared to non-targeting control siRNA pool) in BxPC-3 and Hs700T cells. (**C**) Cell proliferation/viability assayed (by WST-1) in BxPC-3 cells following SMURF1 siRNA-mediated knockdown (compared to non-targeting control). Assay done in triplicate (mean +/− 1SD shown); *, *P*<0.05 (Student's t-test). (**D**) Cell proliferation/viability assayed in Hs700T cells, as above. (**E**) Cell invasion assayed (by Boyden chamber) in BxPC-3 cells following SMURF1 knockdown (compared to non-targeting control). Assay done in triplicate (mean +/− 1SD shown). Note, the reduced proliferation observed with SMURF1 knockdown in Hs700T cells precluded an assay of cell invasion (where invaded cell numbers at 72 hrs are influenced by doubling times).

We also sought to evaluate the effect of siRNA knockdown in other pancreatic cancer cell lines. We chose two cell lines, BxPC-3 cells which (like AsPC-1 cells and most pancreatic tumors) have mutated *SMAD4* (here by homozygous deletion) [21], and Hs700T cells which are wildtype for *SMAD4* and have an intact TGFβ growth-inhibitory pathway (Fig. S1). Notably, neither of these lines harbors focal amplification of *SMURF1* (AsPC-1 cells are the only established line with focal amplification), nor elevated SMURF1 protein levels (Fig. 4A). Knockdown of SMURF1 (validated by Western blot; Fig. 4B) led to modestly reduced cell proliferation in BxPC-3 cells (Fig. 4C), and more so in TGFβ-growth inhibitory pathway-intact Hs700T cells (Fig. 4D). SMURF1 knockdown also resulted in reduced cell invasion in BxPC-3 cells (Fig. 4E), though not significantly so. However, given that *SMURF1* is neither focally amplified nor overexpressed in these lines, a simple explanation for the discordant phenotypes (compared to AsPC-1) is that SMURF1 may not function as an oncogenic driver in these cell contexts.

**Figure 5:**
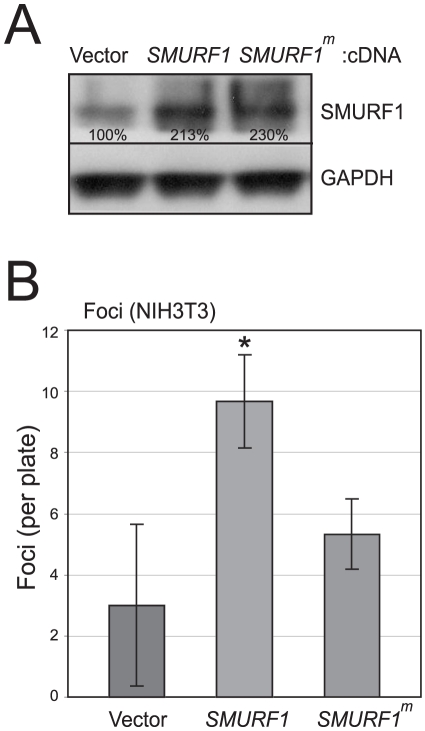
SMURF1 overexpression in NIH-3T3 cells leads to loss of contact inhibition. (**A**) Transfection of SMURF1 cDNA, or a catalytic mutant of SMURF1 (C699A) (SMURF1^m^), leads to overexpression (by Western blot) compared to empty vector control. SMURF1 overexpression levels, normalized to GAPDH, are indicated. (**B**) SMURF1 (but not SMURF1^m^) overexpression in NIH-3T3 cells leads to loss of contact inhibition (i.e. increased foci formation). Assays done in triplicate (mean +/− 1SD shown); *, *P*<0.05 (Student's t-test).

Finally, in complementary, overexpression studies, we transfected *SMURF1* cDNA (expressed from a CMV promoter) into NIH-3T3 mouse fibroblasts. Overexpression of SMURF1, confirmed by Western blot (Fig. 5A), led to a significant loss of contact inhibition (i.e. increased foci), compared to a vector control (Fig. 5B). Notably, transfection of a catalytically-inactive mutant of SMURF1 (C699A) [22] did not reduce contact inhibition (Fig. 4B), indicating that this oncogenic activity is dependent on the E3 ubiquitin protein ligase activity of SMURF1.

## Discussion

Here, we set out to pinpoint and discover the oncogene driving 7q21-q22 amplification in pancreatic cancer. High-resolution genomic profiling of pancreatic cancer cell lines and early-passage xenografts defined a 325 Kb minimal amplicon spanning *SMURF1*. Transcript levels of *SMURF1* were elevated in specimens with gain/amplification, and by FISH we confirmed *SMURF1* amplification in primary pancreatic cancers. Using complementary approaches of knockdown (in focally-amplified AsPC-1 cells) and overexpression (in NIH-3T3 cells), we determined that *SMURF1* amplification/overexpression does not alter cell proliferation, but promotes cell invasion, anchorage-independent growth, and loss of contact inhibition, of which at least the latter is dependent on its catalytic activity.

SMURF1 was initially an intriguing oncogene candidate because of its known connection to TGFβ signaling. The TGFβ pathway, at least early in tumor development, is growth suppressive [23]. Normally, TGFβ binds to its receptors (TGFβRI, TGFβRII), leading to the phosphorylation of signal transducers SMAD2/SMAD3, which then shuttle to the nucleus and in complex with SMAD4 mediate transcription. Key transcriptional responses include induction of *CDKN2B* (p15Ink4b) and *CDKN1A* (p21Cip1), and repression of *MYC*, together leading to G_1_ cell-cycle arrest. The TGFβ growth suppressive pathway is commonly disrupted in pancreatic cancer, most often through mutation/deletion of *SMAD4*, but also through inactivation/loss of *TGFβRI* and *TGFβRII* [4].

SMURF1 is a HECT-domain E3 ubiquitin ligase (E3 ubiquitin ligases carry out the third and substrate-specific step in protein ubiquitination). SMURF1 promotes the nuclear export of TGFβ pathway inhibitor SMAD7 (increasing its availability), and the destruction of TGFβRI and SMAD4 (through ubiquitination-mediated degradation) [15], [16]. All these activities should serve to antagonize TGFβ signaling, and together provide a strong rationale for *SMURF1* amplification/overexpression in pancreatic cancer. It was notable then, that SMURF1 knockdown in AsPC-1 cells did not enhance TGFβ-pathway transcription (though perhaps not surprising, given the inactivating mutation of *SMAD4*). Therefore, the oncogenic activities of SMURF1 must act at least in part independently of its functions in TGFβ signaling (at least at the pathway level of SMAD4). To this end, SMURF1 has also been shown to dissolve tight junctions (by degradation of RhoA) during epithelial-mesenchymal transition [24], and focal adhesions (by degradation of talin heads) to potentiate cell migration [25]. Additional studies should clarify the key SMURF1 substrates linked to invasiveness and anchorage-independent growth in pancreatic cancers with 7q22 amplification.

During the progress of this work, two other studies characterized the 7q21-q22 amplicon in pancreatic cancer. Suzuki *et al.* [26] by genomic profiling of cell lines identified the amplicon in AsPC-1 cells, with the amplicon peak spanning 11 genes. Further efforts focused on two genes, *TRRAP* and *SMURF1*, with significantly elevated expression when amplified. However, in contrast to our study, they reported that knockdown of SMURF1 inhibited AsPC-1 cell proliferation. Notably, though, they evaluated only one siRNA. Given our results that four independent siRNAs knocked down SMURF1 levels comparably and decreased invasion without affecting cell proliferation, we suggest that their finding might reflect a non-specific or specific off-target RNAi effect. Indeed, growth inhibition is a common non-specific effect, triggered by a type I interferon response to siRNA [27]. Suzuki *et al.* went on to show that SMURF1 overexpression in two pancreatic cancer cell lines enhanced colony growth on tissue culture plastic. Nonetheless, our findings based on knockdown in the physiologically-relevant context of focal *SMURF1* amplification suggest that the main oncogenic function of SMURF1 relates to promoting cell invasiveness rather than proliferation.

In another recent study, Laurila *et al.* [28] by FISH analysis of cell lines delimited the 7q21-q22 amplicon to 0.77 Mb spanning 10 genes (including *SMURF1*), but focused their efforts on *ARPC1A* and *ARPC1B*, subunits of the Arp2/3 complex functioning in actin polymerization. Using RNAi, they found that knockdown of either reduced cell motility, and knockdown of *ARPC1A* also reduced cell invasion. Though our minimal amplicon excluded *ARPC1A* and *ARPC1B*, it is nonetheless possible that their amplification contributes to motility/invasion in tumors where they are amplified. It is not uncommon to find multiple driver oncogenes within tumor amplicons (e.g. ref. [29]). Indeed, our own studies do not resolve whether *KPNA7*, within our 325 Kb minimal amplicon, might also have an oncogenic role (along with *SMURF1*).

To summarize, by genomic profiling and functional analysis we identified *SMURF1* as an amplified oncogene driving cell invasiveness in pancreatic cancer. Perhaps of most significance, as an enzyme SMURF1 represents a tractable drug target. Other E3 ubiquitin ligases have been linked to cancer, and because of their substrate specificity E3 ubiquitin ligases are thought to be attractive targets for therapy [30]. Indeed, several small molecule inhibitors (including against MDM2, a regulator of TP53) are presently being evaluated [31]. Our findings identify SMURF1 as a possible new target for molecularly-directed therapy against the devastating disease of pancreatic cancer.

## Materials and Methods

### Specimens

Pancreatic cancer cell lines, described previously [12], and NIH-3T3 cells were obtained from the American Type Culture Collection (Manassas, VA). Pancreatic cancer xenografts, which effectively enrich the tumor epithelial fraction for DNA analysis, were generated as described [32] at the Johns Hopkins Hospital, with approval from the Institutional Review Board (IRB) (protocol ID 05-04-14-02) and Animal Care and Use Committee (protocol ID MO05M466). Briefly, a 1 mm^3^ piece of the primary tumor was soaked in Matrigel (Collaborative Biomedical Research), then implanted subcutaneously in a nu/nu mouse. Engrafted tumors were harvested when they reached 1–2 cm in diameter, and tumor cell enrichment confirmed by H&E-stained frozen section. DNA and RNA were isolated using the Qiagen (Valencia, CA) AllPrep kit. Eleven of the 48 xenografts were previously profiled by lower-resolution CGH on cDNA arrays [6].

### Array CGH

CGH was done using Agilent (Santa Clara, CA) catalogue 244K CGH arrays. DNAs were labeled as described [33], then hybridized (*vs.* a pool of eight sex-matched normal leukocyte DNAs) following the manufacturer's instructions. Arrays were scanned using an Agilent G2505B scanner, and data extracted and normalized using Agilent Feature Extraction software (version 9.1) with default settings. Copy number alterations were called using cghFLasso [34], and low-level gains and higher-level amplifications defined by cghFLasso tumor/normal ratios >1.3 and >3.0, respectively. CGH data detailed herein are available at GEO (GSE19852); a complete description of the dataset is in preparation.

### Expression profiling

Expression profiling was done using Agilent catalogue 44K Whole Human Genome arrays. RNAs were labeled using the Quick Amp Labeling kit (Agilent), then hybridized (*vs.* a universal RNA reference pool of 11 cancer cell lines [35]) following the manufacturer's instructions. After scanning and data extraction (as above), expression data were normalized (mean-centered) by array and by gene, and mean-centered log_2_ ratios reported.

### FISH

A tissue microarray containing 105 pancreatic ductal adenocarcinoma cases (archived at Stanford University, and used with IRB approval) was previously described [6]. Probe labeling and FISH were performed using Vysis (Downers Grove, Illinois) reagents according to the manufacturer's protocols. A locus-specific BAC mapping to *SMURF1* at 7q22.1 (RP11-62N3; BACPAC Resources Centre, Oakland, CA) was labeled with SpectrumOrange, and co-hybridized with SpectrumGreen-labeled chr 7 centromere probe (CEP7; Vysis). Slides were counterstained with DAPI, and imaged using an Olympus BX51 fluorescence microscope with Applied Imaging (San Jose, CA) Cytovision 3.0 software. Twenty-five tumor cells were scored per case, and amplification defined as an average *SMURF1*/CEP7 ratio >2.5.

### siRNA transfections

On-TARGETplus siRNAs targeting *SMURF1*, along with a negative control siRNA pool (ON-TARGETplus siCONTROL Non-targeting Pool), were obtained from Dharmacon (Lafayette, CO). Sequences of siRNAs are listed in Table S1. AsPC-1 cells were grown at 37°C in complete media of RPMI-1640 (Invitrogen, Carlsbad, CA), 10% FBS, 50 U/ml penicillin, and 50 U/ml streptomycin. For transfection, 150,000 cells were seeded per 6-well plate well, and transfected using Lipofectamine 2000 reagent (Invitrogen) according to the manufacturer's protocol. Cells were transfected with a final concentration of 50 nM siRNA for 6 hrs.

### Western blot

Cells were lysed in 1× RIPA buffer supplemented as described [6]. Forty µg total protein lysate was electrophoresed on a 4–15% polyacrylamide gel, then transferred to PVDF membrane and blocked in TBST-T with 5% dry milk. Antibodies were used as follows: anti-SMURF1 rabbit polyclonal antibody (H-60; Santa Cruz Biotechnology, Santa Cruz, CA) at 1∶500 dilution; anti-GAPDH rabbit polyclonal antibody (Santa Cruz Biotechnology) at 1∶5,000 dilution; HRP-conjugated anti-rabbit IgG (Pierce, Rockford, IL) at 1∶20,000 dilution. Detection was done using an ECL kit (GE Healthcare, Piscataway, NJ), and intensities quantified by densitometry using Scion Image software (Scion Corporation, Fredrick, MD).

### Cell growth and invasion assays

Cell proliferation/viability was quantified by colorimetry based on the metabolic cleavage of the tetrazolium salt WST-1 in viable cells, according to the manufacturer's protocol (Roche, Indianapolis, IN). BrdU incorporation was determined by colorimetric ELISA using the BrdU Cell Proliferation Assay, according to the manufacturer's protocol (Cell Signaling Technology, Danvers, MA). Invasion was quantified by Boyden chamber assay (BD Biosciences, San Jose, CA). Briefly, 24 hrs after transfection, 50,000 cells were plated into 24-well Matrigel-coated inserts with a 0.5% to 10% FBS gradient. Seventy-two hrs later, cells were fixed, stained with crystal violet, and cells traversing the membrane counted. All assays were done as triplicate transfections, and all experiments were repeated at least once with similar results.

### TGFβ transcriptional reporter assay

Cells were co-transfected with 4 µg p3TP-Lux (Addgene, Cambridge, MA), a TGFβ responsive firefly luciferase reporter containing three consecutive TPA response elements (TREs) and a portion of the plasminogen activator inhibitor 1 (PAI-1) promoter region [17], along with 0.4 µg pRL-TK (Promega, Madison, WI) expressing Renilla luciferase as an internal normalization control. Luciferase activity was assayed 48 hrs after transfection (by Lipofectamine 2000) using the dual luciferase reporter assay system (Promega, Madison, WI). Reporter activity is expressed as the ratio of firefly/Renilla. Assays were done as triplicate transfections, and repeated at least once with similar results.

### Anchorage-independent growth

A pGIPZ shRNAmir construct targeting *SMURF1* (V2LHS_229724), along with a non-targeting pGIPZ shRNAmir control, were obtained from Open Biosystems (Huntsville, AL). To create stably-transduced AsPC-1 cell pools, lentiviral constructs (along with Trans-lentiviral packaging mix plasmids) were transfected into 293TN producer cells (System Biosciences, Mountain View, CA), and supernatant packaged virus transduced into AsPC-1 cells following the manufacturer's instructions (Open Biosystems' Trans-lentiviral GIPZ packaging protocol). Two days post-infection, 1 µg/ml puromycin (Invitrogen) was added to the culture medium, and cells selected for 14 days. Anchorage independent growth was assayed by colony formation in soft agar. Briefly, 10,000 cells were embedded in a 6-well plate well within a top layer of 0.36% agarose in complete media, over a layer of 0.48% agarose in complete media. Cells were grown for 14–21 days, then visible colonies counted after staining with 0.015% Neutral Red solution. Assays were done as triplicate transductions, and repeated at least once with similar results.

### NIH-3T3 focus formation assay

Full-length human *SMURF1* cDNA expression vector, pcDNA3.1-SMURF1 was a kind gift from Di Chen (University of Rochester Medical Center, Rochester, NY), and the parent vector pcDNA3.1 was purchased from Invitrogen (Carlsbad, CA). A catalytic mutant SMURF1 (C699A) [22] was engineered using the QuickChange XL II Site-Directed Mutagenesis Kit from Stratagene (La Jolla, CA), with the following mutagenic primers: 5′-CGTGGAGGAGACC**GCC**GGGTTTGCTGTGG -3′ (degenerate, mutated bases denoted by bold text) and 5′-CCACAGCAAACCC**GGC**GGTCTCCTCCACG-3′. Fifty thousand cells were seeded per 60 mm plate, and 8 µg of plasmid was transfected by Lipofectamine 2000 reagent (Invitrogen) according to the manufacturer's protocol. Two days after transfection, cells from each 60 mm plate were re-plated into two 10 cm plates and grown to confluence over 28 days. Visible foci were counted after methanol fixation and Giemsa staining. Assays were done as triplicate transfections, and repeated at least once with similar results.

## Supporting Information

Table S1 **siRNA sequences targeting SMURF1.**
(PDF)

Figure S1 **Hs700T cells display TGFβ-induced growth inhibition.** Hs700T cells were plated, and then 2 ng/ml TGFβ (or vehicle control) added and cell proliferation/viability assayed (by WST-1) daily. Assays were done in triplicate (mean +/− 1SD shown); *, *P*<0.05; **, *P*<0.01; ***, *P*<0.001 (Student's t-test). Consistent with intact TGFβ growth inhibition, no deletions spanning *SMAD4* (244K Agilent CGH array data; not shown) and no point mutations of *SMAD4* (Illumina RNAseq analysis; not shown) were identified.
(EPS)
