# Supplementary material for: SMURF1 Amplification Promotes Invasiveness in Pancreatic Cancer
Source: PLoS One. 2011 Aug 22;6(8):e23924. doi: 10.1371/journal.pone.0023924 (PMC3161761; doi:10.1371/journal.pone.0023924)
Supplement: Table S1 — siRNA sequences targeting SMURF1. (PDF) [file pone.0023924.s001.pdf]

Table S1. siRNA sequences targeting *SMURF1*

**Name**	**Sequence**
SMURF1 #1	5'-GCACUAUGAUCUAUAUGUU-3'
SMURF1 #2	5'-GGAGGAGACCUGCGGGUUU-3'
SMURF1 #3	5'-GAUCGACAUUCCACCAUAU-3'
SMURF1 #4	5'-AAGAAUACGUCCGGUUGUA-3'
